# Persistent high sepsis-induced coagulopathy and sequential organ failure assessment scores can predict the 28-day mortality of patients with sepsis: A prospective study

**DOI:** 10.1186/s12879-024-09154-x

**Published:** 2024-03-04

**Authors:** Junyu Li, Huizhen Liu, Na Wang, Fengrong Wang, Na Shang, Shubin Guo, Guodong Wang

**Affiliations:** 1 Department of Emergency Medicine, Beijing Chao-Yang Hospital, Beijing Key Laboratory of Cardiopulmonary Cerebral Resuscitation, Capital Medical University, Beijing, China; 2 Department of Emergency Medicine, Capital Medical University School of Rehabilitation Medicine, Beijing Bo’Ai Hospital, China Rehabilitation Research Center , Beijing, China; 3 Cardiovascular Department, Capital Medical University School of Rehabilitation Medicine, Beijing Bo’Ai Hospital, China Rehabilitation Research Center, Beijing, China

**Keywords:** Sepsis, Sepsis-induced coagulopathy, Sequential organ failure assessment score, Time course, Prognosis

## Abstract

**Background:**

The performance of the sepsis-induced coagulopathy (SIC) and sequential organ failure assessment (SOFA) scores in predicting the prognoses of patients with sepsis has been validated. This study aimed to investigate the time course of SIC and SOFA scores and their association with outcomes in patients with sepsis.

**Methods:**

This prospective study enrolled 209 patients with sepsis admitted to the emergency department. The SIC and SOFA scores of the patients were assessed on days 1, 2, and 4. Patients were categorized into survivor or non-survivor groups based on their 28-day survival. We conducted a generalized estimating equation analysis to evaluate the time course of SIC and SOFA scores and the corresponding differences between the two groups. The predictive value of SIC and SOFA scores at different time points for sepsis prognosis was evaluated.

**Results:**

In the non-survivor group, SIC and SOFA scores gradually increased during the first 4 days (*P* < 0.05). In the survivor group, the SIC and SOFA scores on day 2 were significantly higher than those on day 1 (*P* < 0.05); however, they decreased on day 4, dropping below the levels observed on day 1 (*P* < 0.05). The non-survivors showed higher SIC scores on days 2 (*P* < 0.05) and 4 (*P* < 0.001) than the survivors, whereas no significant differences were found between the two groups on day 1 (*P* > 0.05). The performance of SIC scores on day 4 for predicting mortality was more accurate than that on day 2, with areas under the curve of 0.749 (95% confidence interval [CI]: 0.674–0.823), and 0.601 (95% CI: 0.524–0.679), respectively. The SIC scores demonstrated comparable predictive accuracy for 28-day mortality to the SOFA scores on days 2 and 4. Cox proportional hazards models indicated that SIC on day 4 (hazard ratio [HR] = 3.736; 95% CI: 2.025–6.891) was an independent risk factor for 28-day mortality.

**Conclusions:**

The time course of SIC and SOFA scores differed between surviving and non-surviving patients with sepsis, and persistent high SIC and SOFA scores can predict 28-day mortality.

## Background

Sepsis is defined as “life-threatening organ dysfunction caused by a dysregulated host response to infection” [1]. Despite a decrease in mortality among patients with sepsis due to the promotion of the “Surviving Sepsis Campaign,” sepsis remains a major cause of death worldwide [2]. Recent studies have shown that the mortality rate of sepsis is 40% in the intensive care unit (ICU) and 26% in all hospitals [3]. The evaluation and prediction of the prognoses of patients with sepsis are crucial for early stratification and accurate treatment. Coagulopathy may be experienced by 50–70% of patients with sepsis, which ranges from mild thrombocytopenia to disseminated intravascular coagulation (DIC) [4–5]. In patients with sepsis, coagulopathy causes organ dysfunction due to microvascular thrombosis and is related to unfavorable prognoses [6].

Currently, sepsis-associated coagulopathy is mostly evaluated using scoring systems, such as the International Society on Thrombosis and Hemostasis (ISTH) overt-DIC criteria, the Japanese Association for Acute Medicine (JAAM) DIC criteria, and the Japanese Society on Thrombosis and Hemostasis DIC criteria [7–9]. However, these diagnostic scoring systems are either too intricate or too focused on various underlying diseases rather than specifically on sepsis, and the pathophysiology of coagulopathy differs between diseases. For example, suppressed fibrinolysis is significant in sepsis-associated coagulopathy due to increased plasminogen activator inhibitor-1 levels, which result in hypercoagulation and unfavorable microthrombosis [10]. The JAAM DIC criteria are more specific for sepsis and have been reported to be valuable in predicting poor prognoses for patients with sepsis [11] and recognizing appropriate candidates for anticoagulant therapy [12]. However, as one of the 4 items included in the JAAM DIC criteria [8], systemic inflammatory response syndrome has been replaced by sequential organ failure assessment (SOFA) score in the new sepsis definition [1]. Therefore, in 2017, the ISTH proposed sepsis-induced coagulopathy (SIC) diagnostic criteria specifically for sepsis based on SOFA score [13] to identify septic coagulopathy early and direct anticoagulation treatment. The SOFA score was used to confirm the presence of sepsis, not to reflect the severity; therefore, the score for SOFA was limited to two points even if the SOFA score was more than two [14]. Although the SIC criteria are relatively simple compared to the JAAM DIC criteria, they are comparable concerning mortality prediction [15].

The prognostic power of the SIC scoring system for patients with sepsis has been evaluated recently; however, the results have been inconsistent. Lu et al. [16] found that the SIC score could predict both 7-day and 28-day mortality in patients with sepsis in the ICU. However, another study reported that the SIC score was an independent risk factor for in-hospital mortality in patients with septic shock rather than in other patients with sepsis [17]. According to Schmoch et al. [18], SIC is associated with significantly higher 28-day, 90-day, 180-day, and ICU mortality in patients with sepsis rather than in those with septic shock. Some experts suggest that the coagulation dysfunction in patients with sepsis is a dynamic and evolving process [19]. Park et al. [20] found that among septic patients, the mean ISTH-DIC scores on day 1 and day 3 were 4.0 ± 1.2 and 4.0 ± 1.3, respectively; however, after grouping patients according to hospital mortality, the mean DIC scores on day 1 and day 3 were 3.8 ± 1.1 and 3.6 ± 1.1 respectively in survivors, and 4.3 ± 1.3 and 4.8 ± 1.5 respectively in non-survivors. Similarly, the trend of platelet changes varies among patients with different prognoses [21]. SOFA score was proposed to assess the severity of organ dysfunction in critically ill patients [22]. Extensive studies have been conducted on the importance of SOFA scores in predicting the prognoses of patients with sepsis, and the findings revealed that SOFA scores demonstrated strong predictive power in assessing mortality [23–25].

Although many studies have focused on the performance of SIC and SOFA scores in evaluating the prognoses of patients with sepsis, few have examined how they changed over time. Therefore, this study aimed to investigate the time course of SIC and SOFA scores and their association with outcomes in patients with sepsis. The changes in coagulation function and organ dysfunction may be more pronounced in the early stages of the disease, so we planned to evaluate the SIC and SOFA scores on days 1, 2, and 4 [15].

## Methods

### Study design and population

This was a single-center, prospective, observational study. Patients with sepsis admitted to the emergency department of the China Rehabilitation Research Center (Beijing, China), a university-affiliated tertiary hospital, between December 2018 and November 2021 were included in this study. The exclusion criteria were as follows: age < 18 years; pregnancy; history of hematopoietic malignancy; history of serious liver disease; history of thrombocytopenia and coagulopathy; and treatment with radiotherapy, chemotherapy, or warfarin. Patients with incomplete SIC assessment data and those who died within 24 h of admission were also excluded after enrollment. The Institutional Review Board of the China Rehabilitation Research Center (2018-061-1) approved this study. All participants or their legal representatives signed an informed consent form, and this study was conducted following the Declaration of Helsinki.

### Data collection

The demographic data and clinical characteristics of the participants, including age, sex, body mass index (BMI), and comorbidities, were collected upon admission. Additionally, routine laboratory test results and Acute Physiology and Chronic Health Evaluation II (APACHE II) scores were evaluated and recorded 24 h after admission. The most abnormal result was recorded if multiple results were available within the first 24 h. The SOFA scores, platelet counts and prothrombin time-international normalized ratios (PT-INRs) were evaluated on days 1, 2, and 4. Platelet counts were measured using the Mindray BC-5390 automated hematology analyzer (Mindray, Shenzhen, China), and PT-INRs were determined using the Sysmex CA-7000 automated coagulation analyzer (Sysmex, Chuo-ku, Japan). All patients received standard treatment strategies according to the instructions of the Surviving Sepsis Campaign Guideline [26]. The primary outcome was 28-day mortality, and all participants were categorized into either survivor or non-survivor groups.

### Definitions

Sepsis was defined as an increase in the SOFA score of at least two points caused by current infection, according to the Sepsis-3 definition [1]. Septic shock was defined as fluid-resistant hypotension (mean arterial pressure less than 65 mmHg) requiring vasopressors with a serum lactate level > 2 mmol/L [1]. SIC was diagnosed according to the criteria proposed by members of the ISTH in 2017 [13]. The SIC scoring system comprises the following three factors: PT-INR, platelet count, and SOFA score (composed of respiratory, cardiovascular, hepatic, and renal SOFA). The maximum total score was six; a total score of four or more was defined as SIC (Table 1) [13].

**Table 1:** The sepsis-induced coagulopathy scoring system

Category	Parameter	0 point	1 point	2 points
Prothrombin time	PT-INR	≤ 1.2	> 1.2	> 1.4
Coagulation	Platelet count (×10^9^/L)	≥ 150	< 150	< 100
Total SOFA	SOFA four items	0	1	≥ 2

A total score of four or more is defined as SIC. The total SOFA score is the sum of the four items (respiratory, cardiovascular, hepatic, and renal SOFA). PT-INR: prothrombin time-international, SOFA: sequential organ failure assessment.

### Statistical analysis

Normally distributed variables are expressed as means ± standard deviation, and Student’s *t*-tests were used to compare differences between groups. Non-normally distributed variables are expressed as median with interquartile range, and Mann–Whitney *U* tests were used for comparisons between groups. Qualitative data are presented as counts and percentages, and chi-square tests were used for difference comparisons. Expectation-maximization algorithm was used to impute the quantitative variables if missing data were less than 20%, while there were no qualitative variables with missing data in the current study. A generalized estimating equation analysis (with autocorrelation as the working correlation matrix) was conducted to evaluate the differences between SIC scores at various time points, the differences between SIC scores in the two groups, and the interaction effect between groups and various time points. If the interaction effect was statistically significant, a simple-effect analysis was performed. The trend of changes in SOFA scores in different groups was also analyzed using the same method. We plotted the receiver operating characteristic (ROC) curves and calculated the area under the ROC curve (AUC) to evaluate the predictive value of SIC scores and SOFA scores at various time points for the prognosis of sepsis. Introducing those variables related to 28-day mortality in the univariable analysis, forward stepwise multivariate Cox proportional hazards analyses were performed to determine the factors independently associated with the 28-day survival of patients with sepsis. Therefore, patients were determined to have SIC based on the SIC scores on days 2 and 4. The variables that were introduced in Model 1 were age, D-dimer, albumin, and lactate levels, SOFA score, APACHE II score, septic shock, and SIC on day 2. The variables that were introduced in Model 2 were age, D-dimer, albumin, and lactate levels, SOFA score, APACHE II score, septic shock, and SIC on day 4. A two-sided *P* < 0.05 was considered statistically significant. SPSS (version 26.0; IBM, Armonk, NY, USA), MedCalc (version 19.3; MedCalc Software Ltd, Ostend, Belgium) and GraphPad Prism (version 9.4; GraphPad Software, San Diego, CA, USA) were used to perform the analyses and draw the figures.

## Results

### Patient characteristics

In this study, 225 consecutive patients with sepsis were enrolled, of whom two with incomplete data and 14 who died within 24 h of admission were excluded. Ultimately, 209 participants were included in the analysis. The median age of the patients was 83 years (74, 88), and 60.3% (126/209) were male. The 28-day mortality rate was 37.3% (78/209). The main infected sites were the lungs (78.9%), urinary tract (24.4%), abdomen (22.0%), and soft tissues (5.3%).

Table 2 presents the baseline clinical characteristics and laboratory data of the study participants. The non-survivors were older and had more patients with septic shock than the survivors. No significant differences in sex, BMIs, comorbidities, or sites of infection were found between the two groups. Moreover, significantly higher D-dimer and lactate levels were observed in the non-survivors. However, albumin concentrations were found to be lower in the non-survivors. Furthermore, non-survivors had significantly higher APACHE II scores on admission. The length of stay of the survivors was longer than that of the non-survivors.

**Table 2:** Baseline characteristics of the study population

Characteristic	Overall (*n* = 209)	Survivors (*n* = 131)	Non-survivors (*n* = 78)	P-value
Age (years), median (IQR)	83 (74, 88)	81 (70, 88)	85 (80, 90)	0.002
Male, n (%)	126 (60.3%)	83 (63.4)	43 (55.1)	0.240
BMI (kg/m^2^), mean ± SD	21.73 ± 4.31	22.18 ± 4.20	20.99 ± 4.41	0.053
**Comorbidities, n (%)**				
Hypertension	126 (60.3%)	78 (59.5%)	48 (61.5%)	0.775
Diabetes mellitus	91 (43.5%)	54 (41.2%)	37 (47.4%)	0.381
Cardiovascular disease	66 (31.6%)	40 (30.5%)	26 (33.3%)	0.674
Cerebrovascular disease	62 (29.7%)	38 (29.0%)	24 (30.8%)	0.787
Chronic kidney disease	13 (6.2%)	7 (5.3%)	6 (7.7%)	0.701
**Site of infection, n (%)**				
Lung	165 (78.9%)	99 (75.6%)	66 (84.6%)	0.121
Abdomen	46 (22.0%)	32 (24.4%)	14 (17.9)	0.274
Urinary tract	51 (24.4%)	32 (24.4%)	19 (24.4%)	0.991
Soft tissue	11 (5.3%)	4 (3.1%)	7 (9.0%)	0.125
**Laboratory values**				
WBC (×10^9^/L), median (IQR)	13.27 (9.29, 19.45)	13.10 (8.90, 18.55)	13.91 (9.92, 20.22)	0.414
CRP (mg/L), median (IQR)	99.69 (30.07, 207.08)	103.40 (23.80, 220.00)	82.24 (39.82, 186.92)	0.714
IL-6 (pg/mL), median (IQR)	102.90 (35.90, 466.70)	92.00 (34.20, 339.10)	128.70 (39.50, 508.35)	0.333
Platelet (×10^9^/L), median (IQR)	167.00 (110.00, 249.00)	165.00 (112.00, 243.00)	174.00 (108.00, 250.25)	0.725
PT (s), median (IQR)	13.20 (12.30, 14.20)	13.00 (12.30, 14.10)	13.35 (12.20, 15.00)	0.126
INR, median (IQR)	1.16 (1.07, 1.25)	1.14 (1.07, 1.24)	1.19 (1.06, 1.33)	0.111
APTT (s), median (IQR)	34.20 (28.60, 42.40)	33.40 (28.60, 41.70)	35.20 (28.53, 43.60)	0.497
Fibrinogen (g/L), median (IQR)	4.52 (3.23, 5.46)	4.81 (3.27, 5.46)	4.06 (2.94, 5.31)	0.089
D-dimer (mg/L), median (IQR)	2.74 (1.34, 6.28)	2.27 (1.17, 5.13)	4.24 (1.75, 7.88)	0.006
Albumin (g/L), mean ± SD	33.94 ± 6.18	34.96 ± 6.09	32.22 ± 6.00	0.002
Creatinine (umol/L), median (IQR)	137.00 (93.35, 208.50)	131.00 (89.00, 192.00)	175.90 (100.78, 239.75)	0.050
Lactate (mmol/L), median (IQR)	2.52 (1.45, 4.40)	2.20 (1.30, 4.26)	3.01 (1.85, 5.88)	0.009
**APACHE II score, median (IQR)**	24.00 (19.00, 28.00)	21.00 (17.00, 27.00)	27.00 (22.75, 33.00)	< 0.001
**Septic shock, n (%)**	69 (33.0%)	34 (26.0%)	35 (44.9%)	0.005
**Length of stay (days), median (IQR)**	13.00 (8.00, 24.00)	17.00(10.00, 32.00)	7.50 (3.00, 15.25)	< 0.001

BMI: body mass index; WBC: white blood cell; CRP: C-reactive protein; IL-6: interleukin-6; PT: prothrombin time; INR: international normalized ratio; APTT: activated partial thromboplastin time; APACHE II: Acute Physiology and Chronic Health Evaluation II; IQR: interquartile range; SD: standard deviation

### Time course of sepsis-induced coagulopathy scores

**Fig. 1:**
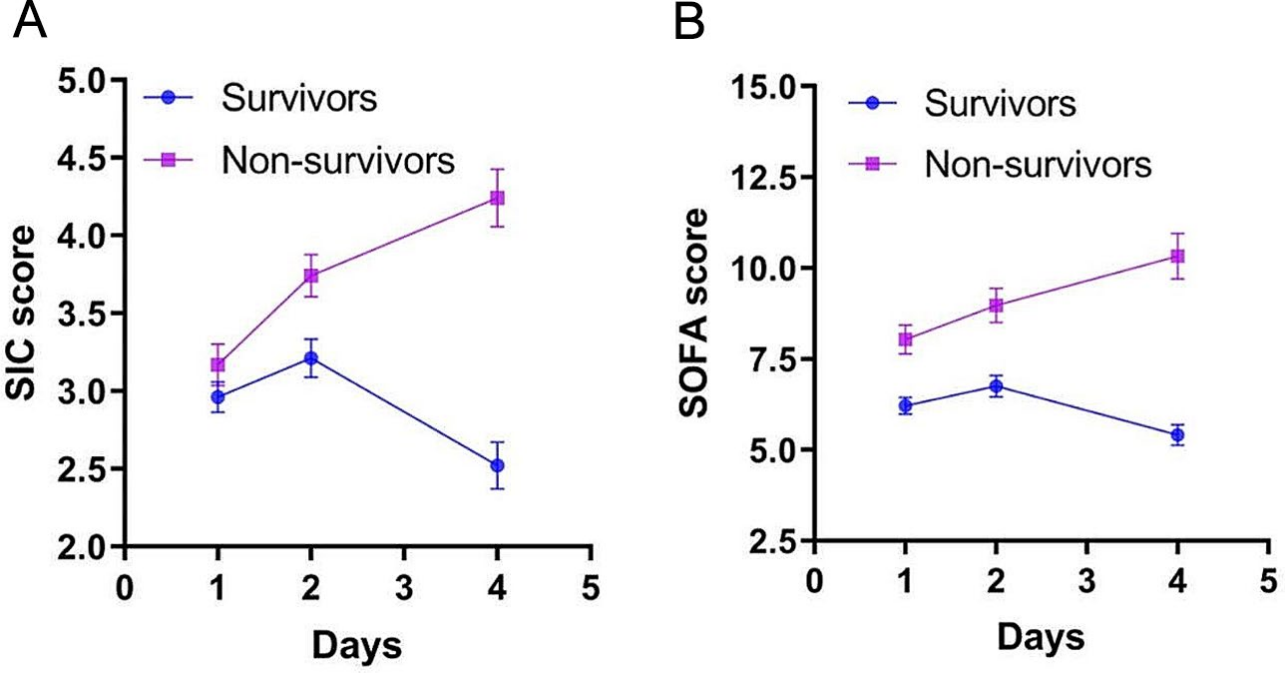
SIC scores and SOFA scores at different time points in survivors and non-survivors. (A) SIC scores; (B) SOFA scores. SIC: sepsis-induced coagulopathy; SOFA: Sequential Organ Failure Assessment

The generalized estimating equation model indicated that the main effect of group (Wald *χ2* = 23.151, *P* < 0.001), the main effect of time (Wald *χ2* = 33.427, *P* < 0.001), and the interaction effect between group and time (Wald *χ2* = 55.008, *P* < 0.001) were all significant. Further simple-effect analysis revealed that the SIC scores of the non-survivors gradually increased during the first 4 days (*P* < 0.001). Among the survivors, the SIC scores on day 2 were significantly higher than those on day 1 (*P* < 0.05); however, they began to decrease on day 4, dropping below the levels observed on day 1 (*P* < 0.001). The non-survivors showed higher SIC scores on days 2 (*P* < 0.05) and 4 (*P* < 0.001) than the survivors, whereas no significant difference was observed between the two groups on day 1 (*P* > 0.05; Fig. 1.A).

When it comes to the SOFA scores, the main effect of group (Wald *χ2* = 33.434, *P* < 0.001), the main effect of time (Wald *χ2* = 18.062, *P* < 0.001), and the interaction effect between group and time (Wald *χ2* = 42.390, *P* < 0.001) were all significant, too. Among the non-survivors, the SOFA scores continuously increase during the first 4 days (*P* < 0.05). Conversely, among the survivors, the SOFA scores decreased on day 4, dropping below the levels observed on day 1 (*P* < 0.05), following a temporary increase on day 2 (*P* < 0.05). In addition, the SOFA scores of the non-survivors were higher than those of survivors at all three-time points (*P* < 0.001; Fig. 1.B).

### The predictive efficacy of sepsis-induced coagulopathy scores for 28-day mortality

**Fig. 2:**
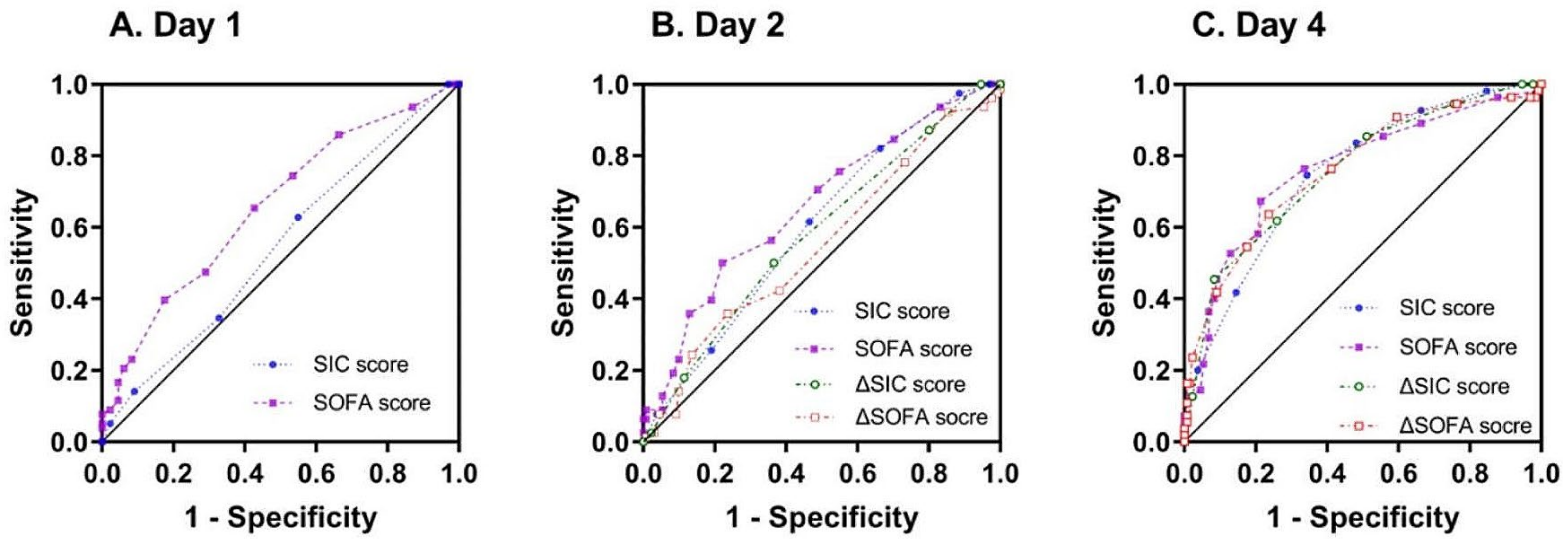
ROC curves of SIC scores and SOFA scores for predicting 28-day mortality. (A) ROC curves of SIC and SOFA scores on day 1 (*n* = 209); (B) ROC curves of SIC and SOFA scores on day 2 and the changes in the scores from days 1 to 2 (*n* = 209); (C) ROC curves of SIC and SOFA scores on day 4 and the changes in the scores from days 1 to 4 (*n* = 186). ROC: receiver operating characteristic; SIC: sepsis-induced coagulopathy; SOFA: Sequential Organ Failure Assessment

The number of non-survivors decreased as the number of days increased. Therefore, the actual number of study participants (survivors/non-survivors) was 209 (131/78), 209 (131/78), and 186 (131/55) on days 1, 2, and 4, respectively. ROC curves of the SIC scores and the SOFA scores at different time points for predicting 28-day mortality were plotted and are shown in Fig. 2. The AUC of the SIC score on day 1 was 0.545 (95% CI: 0.465–0.625; *P* = 0.277), with no statistical significance. However, the AUCs of the SIC score on day 2, ΔSIC score-d2 (the difference between the SIC scores on days 1 and 2), the SIC score on day 4, and ΔSIC score-d4 were 0.601 (95% CI: 0.524–0.679; *P* < 0.05), 0.583 (95% CI: 0.504–0.662; *P* < 0.05), 0.749 (95% CI: 0.674–0.823; *P* < 0.001), and 0.758 (95% CI: 0.682–0.834; *P* < 0.001), respectively. There was no significant difference between the AUC of the SIC and ΔSIC scores at all the time points considered (*P* > 0.05). In addition, the performance of the SIC and ΔSIC scores for predicting 28-day mortality was comparable to that of the SOFA scores on days 2 and 4. However, the SOFA scores performed better than the SIC scores on day 1 (*Z* = 2.479, *P* = 0.013). The cutoff value and the corresponding sensitivity and specificity of SIC scores and SOFA scores for predicting 28-day mortality in patients with sepsis are presented in Table 3.

**Table 3:** Accuracy of SIC and SOFA scores to predict 28-day mortality in patients with sepsis

Variable	Cutoff	Sensitivity	Specificity	AUC (95% CI)
SIC score-d1	-	-	-	0.545 (0.465, 0.625)
SOFA score-d1	6.5	0.654	0.573	0.658 (0.582, 0.734)
SIC score-d2	2.5	0.821	0.336	0.601 (0.524, 0.679)
ΔSIC score-d2	0.5	0.500	0.634	0.583 (0.504, 0.662)
SOFA score-d2	8.5	0.500	0.779	0.660 (0.583, 0.736)
ΔSOFA score-d2	-	-	-	0.552 (0.470, 0.633)
SIC score-d4	3.5	0.745	0.656	0.749 (0.674, 0.823)
ΔSIC score-d4	1.5	0.455	0.916	0.758 (0.682, 0.834)
SOFA score-d4	6.5	0.673	0.786	0.763 (0.684, 0.842)
ΔSOFA score-d4	0.5	0.636	0.763	0.761 (0.683, 0.839)

SIC: sepsis-induced coagulopathy; SOFA: sequential organ failure assessment; ΔSIC score-d2: change in sepsis-induced coagulopathy score between days 1 and 2; ΔSIC score-d4: change in sepsis-induced coagulopathy score between days 1 and 4; ΔSOFA score-d2: change in sequential organ failure assessment score between days 1 and 2; ΔSOFA score-d4: change in sequential organ failure assessment score between days 1 and 4; AUC: area under the curve; CI: confidence interval.

### Predictors for 28-day mortality in patients with sepsis

We categorized the patients into SIC or non-SIC groups according to their SIC scores on days 2 and 4. Multivariate Cox proportional hazards analysis showed that age, albumin levels, APACHE II score, and SIC on day 4 were independent predictors of 28-day mortality in patients with sepsis. However, the SIC on day 2 was not independently associated with patient outcomes (Table 4).

**Table 4:** Predictors of the 28-day mortality in patients with sepsis according to multivariate Cox proportional hazards analysis

	Model 1				Model 2		
	HR	95% CI	*P*-value		HR	95% CI	*P*-value
Age	1.044	1.016–1.072	0.002		1.048	1.015–1.083	0.005
Albumin	0.950	0.918–0.985	0.005		0.940	0.899–0.982	0.005
Lactate	1.080	1.018–1.147	0.011		-	-	-
APACHE II score	1.069	1.039–1.101	< 0.001		1.058	1.021–1.095	0.002
SIC-d2	-	-	-		-	-	-
SIC-d4	-	-	-		3.736	2.025–6.891	< 0.001

Model 1: Cox proportional hazards analysis of the correlation between 28-day mortality and SIC-d2 (*n* = 209).

Model 2: Cox proportional hazards analysis of the correlation between 28-day mortality and SIC-d4 (*n* = 186). APACHE II: Acute Physiology and Chronic Health Evaluation II; SIC: sepsis-induced coagulopathy; HR: hazard ratio; CI: confidence interval.

## Discussion

This study prospectively evaluated the time course of SIC and SOFA scores in patients with sepsis and their association with patient outcomes. The results demonstrated that the trend of changes in SIC and SOFA scores varied among patients with sepsis with different prognoses. We also found that the initial SIC scores were poorly correlated with the prognoses of patients with sepsis, whereas high SIC scores on days 2 and 4 indicated unfavorable outcomes. The SIC scores on day 4 and the ΔSIC scores between days 1 and 4 more accurately predicted 28-day mortality than those on day 2. In addition, the performance of the SIC and ΔSIC scores for predicting 28-day mortality was comparable to the SOFA scores on days 2 and 4; however, the SOFA scores performed better than the SIC scores on day 1.

Sepsis is a serious condition with the high morbidity and high mortality [2, 27] and the prediction of the prognoses of patients with sepsis plays a critical role in better clinical decision-making. Our study indicated that an increased SIC score was significantly related to mortality risk in patients with sepsis, which is consistent with the findings of previous studies. One retrospective study that used a large dataset comprising 9,432 participants has shown that compared with those without SIC, patients with SIC have a 52% increase in both 7-day and 28-day mortality in ICU patients with sepsis [16]. Moreover, Ding et al. [28] have found that the SIC score within 24 h of admission is independently associated with ICU mortality in patients with sepsis. However, our study demonstrated that, although the SIC scores on days 2 and 4 were significantly higher in non-survivors than in survivors, no significant differences were detected in SIC scores on day 1 between the two groups. Notably, our study enrolled emergency rather than ICU patients, leading to a relatively early assessment of SIC scores, which may partially explain the poor performance of initial SIC scores that we found. Another recent study has reported that, while no significant difference is found in 28-day mortality between SIC-positive and SIC-negative patients on day 1, those with SIC have significantly higher mortality on day 2 than those without SIC, corroborating our observations [15]. Additionally, we found that the performance of the SIC score on day 4 for predicting mortality was better than that on day 2, with AUCs of 0.749 (95% CI: 0.674–0.823) and 0.601 (95% CI: 0.524–0.679), respectively. Our multivariate analysis showed similar results. SIC on day 4 was an independent risk factor for 28-day mortality after adjusting for age, D-dimer, albumin, and lactate levels, SOFA score, APACHE II score, and septic shock, whereas SIC on day 2 was not independently associated with mortality. Furthermore, in the present study, the SIC scores demonstrated comparable predictive accuracy for 28-day mortality as the SOFA scores on days 2 and 4, although the SOFA scores performed better than the SIC scores on day 1. It has been found that the SOFA score exhibited robust predictive capability in the assessment of mortality in patients with sepsis [23–25], and our study further confirmed it. Additionally, our findings revealed that the SOFA scores on day 4 performed better in predicting the patients’ 28-day mortality than those on days 1 and 2. These results indicate that in order to better evaluate the prognoses of patients with sepsis, we should dynamically monitor the SIC and SOFA scores of the patients, rather than just focusing on the results on the day of the visit. However, it should be noted that this study did not include the patients with history of thrombocytopenia and coagulopathy, therefore, caution should be maintained when generalizing the results of this study to this population.

Additionally, we found that the sensitivity of the SIC score for predicting 28-day mortality was high, particularly on day 2 (0.821), indicating that it could be used to screen serious patients. However, the specificity of SIC scores on day 2 for predicting mortality was low (0.336). Nevertheless, the specificity increased to 0.656 on day 4. Helms et al. [29] have also found that the SIC score had better sensitivity and worse specificity than the ISTH overt DIC and JAAM DIC scores for predicting mortality in patients with septic shock. Although there was no significant difference between the fixed-day SIC scores and the ΔSIC scores (i.e., the changes from the baseline scores) in predicting 28-day mortality in the current study, the specificity of ΔSIC scores was higher at all of the time points considered, compensating for the low specificity of SIC scores. Therefore, combining SIC and ΔSIC scores can better evaluate the prognoses of patients with sepsis in clinical practice.

We observed that the SIC and SOFA scores increased continuously during the first 4 days after admission in non-survivors of sepsis, whereas, in survivors, the scores began to decrease on day 4 after a temporary increase on day 2. Given the definite correlation between organ dysfunction and adverse outcomes of patients with sepsis [6], it is not difficult to understand that the persistent high SOFA scores indicated a poor prognosis. Previous reports have demonstrated wide-ranging crosstalk between hemostasis and inflammation that may contribute to organ dysfunction in patients with sepsis [5, 30]. Considering the relationship between coagulopathy and organ dysfunction, poorer prognoses in patients with persistent high SIC scores seem reasonable. According to a recent study, which was a secondary analysis of two German trials, persistent SIC is related to a higher SOFA score and mortality in patients with sepsis [18]. Although coagulopathy is directly correlated with poor prognoses of patients with sepsis [6], the effectiveness of anticoagulant therapy is still controversial and further research is urgent [31]. Some experts suggested that evaluating the efficacy of anticoagulant therapy based solely on 28-day mortality difference was not adequate, and additional methods to reflect the treatment effect was warranted [31]. Iba et al. found that the ΔSOFA score (i.e., the change in SOFA score between days 1 and 7) was significantly associated with the 28-day mortality in patients with sepsis and DIC, and suggested that the ΔSOFA score could be used as an additional method to evaluate the effectiveness of anticoagulation [32]. Our study also found that ΔSOFA score between days 1 and 4 can predict the 28-day mortality of patients with sepsis. Moreover, we found that the ΔSIC score between days 1 and 4 had good predictive value for 28-day mortality, indicating that the ΔSIC score might be useful as another supplementary endpoint for studies evaluating the efficacy of anticoagulant therapy in patients with sepsis. However, we have to remind that the participants enrolled in the current study were not routinely treated with anticoagulant according to the instructions of the Surviving Sepsis Campaign Guideline [26], and the ΔSIC or ΔSOFA score should be used cautiously as an endpoint to determine the effectiveness of anticoagulation. Until recently, the time course of the SIC scores in patients with sepsis has not been systematically or prospectively studied, which is the main novelty of our research. However, Akca et al. [21] have found that the trend in platelet count changes differs between survivors and non-survivors among critically ill patients. In both survivors and non-survivors, platelet counts gradually decrease over time, reaching a nadir on day 4 and returning to the admission value after 1 week. Platelet counts continue to increase to higher than their initial values by day 9 in the survivors, whereas, in the non-survivors, no subsequent increase is observed. As mentioned above, the platelet count is one of the components of the SIC scoring system.

This study had some limitations. First, this was a small single-center study conducted in a tertiary hospital with generally older patients, which may have led to a selection bias. Therefore, further prospective multicenter studies with larger sample sizes are required to confirm our findings. Second, we did not assess the time course of the JAAM DIC criteria and inflammatory markers, such as C-reactive protein and procalcitonin, and their association with SIC scores was not evaluated. However, to the best of our knowledge, this is the first prospective study to focus on the time course of SIC and SOFA scores and their association with the outcomes of patients with sepsis in the emergency department.

## Conclusions

The time course of SIC and SOFA scores differed between surviving and non-surviving patients with sepsis. The SIC and SOFA scores increased continuously during the first 4 days after admission in non-survivors of sepsis; however, in survivors, the scores began to decrease on day 4 after a temporary increase on day 2. Persistent high SIC and SOFA scores can predict outcomes in patients with sepsis in the emergency department, indicating that the SIC and SOFA scores should be dynamically monitored in clinical practice rather than just focusing on initial levels. Further multicenter prospective studies should focus on the time course of SIC and SOFA scores and their impact on anticoagulant therapy in patients with sepsis.

## Data Availability

The data supporting the findings of this study are available from the corresponding author upon reasonable request.

## Abbreviations

ICU: intensive care unit
DIC: disseminated intravascular coagulation
ISTH: International Society on Thrombosis and Hemostasis
JAAM: Japanese Association for Acute Medicine
SIC: sepsis-induced coagulopathy
BMI: body mass index
APACHE II: Acute Physiology and Chronic Health Evaluation II
SOFA: Sequential Organ Failure Assessment
PT-INR: prothrombin time-international normalized ratio
ROC: receiver operating characteristic
AUC: area under the ROC curve
WBC: white blood cell
CRP: C-reactive protein
IL-6: interleukin-6
APTT: activated partial thromboplastin time
SD: standard deviation
IQR: interquartile range
HR: hazard ratio
CI: confidence interval
